# Dosimetric parameters predict radiation-induced choanal stenosis in patients with nasopharyngeal carcinoma

**DOI:** 10.1186/s13014-020-01512-8

**Published:** 2020-06-05

**Authors:** Hui Chang, Kai Chen, Ya-lan Tao, Fei Han, Wei-jun Ye, Yuan-hong Gao

**Affiliations:** Department of Radiation Oncology, Sun Yat-sen University Cancer Center; State Key Laboratory of Oncology in South China; Collaborative Innovation Center for Cancer Medicine, 651 Dongfeng Road East, Guangzhou, 510060 Guangdong China

**Keywords:** Nasopharyngeal carcinoma, Choanal stenosis, Radiation dose

## Abstract

**Background:**

Radiation-induced choanal stenosis (RICS) severely decreases life quality of patients with nasopharyngeal carcinoma (NPC) and originates from nasal mucositis, which depends on radiation dose. This self-controlled study aimed to find the correlations between dosimetric parameters and RICS.

**Methods:**

Totally 49 NPC patients treated with intensity-modulated radiotherapy from May 2010 to Aug. 2013 and diagnosed with RICS during follow-up were enrolled into this study. Minimum point dose, maximum point dose, mean dose (Dmean), dose covering ≥33% volume (D33), dose covering ≥66% volume (D66), and volume receiving ≥60 Gy (V60) were compared between the nasal cavities with and without RICS, through paired t-test. The parameters with difference would enter receiver operating characteristic analysis to determine their cutoff values. Then predicting abilities of the cutoff values were tested by Chi-square test.

**Result:**

The nasal cavities with RICS appeared to have higher Dmean, D33, D66 and V60, compared with those without RICS (*P* values were 0.014, 0.003, 0.006 and 0.010). Dmean ≥54.22 Gy, D33 ≥ 61.96 Gy, D66 ≥ 46.50 Gy and V60 ≥ 48.13% were demonstrated to be related with a higher risk of RICS.

**Conclusion:**

Dmean, D33, D66 and V60 of nasal cavity might be used as predictors of RICS. Their values needed to be controlled whenever possible, for ameliorating life quality of NPC patients.

## Introduction

Nasopharyngeal carcinoma (NPC) is one of the most common malignancies in South China and mainly managed with radiotherapy [1]. The 5-year overall survival of NPC patients has already reached 86.3%, due to advent of intensity-modulated radiotherapy (IMRT) and concurrent chemotherapy (CCT) [2]. Nevertheless, treatment-related late toxicities are still common, especially in those receiving CCT. The incidence of late toxicities was reported to be as high as 70.8%. And the incidence of grade 3/4 late toxicities was nearly 11.4% [3, 4]. These toxicities are worth paying attention to because of its adverse influences on life quality of long-term survivors.

Radiation-induced choanal stenosis (RICS) is a rare late toxicity observed in only 4.3% of NPC patients. It leads to serious difficulty in breathing through and discharge from nose. For improving these symptoms, transnasal endoscopic surgery is often needed [5–7]. Ku et al. found that RICS originated from severe choanal mucositis [8]. Since degree of head-and-neck mucositis depends on radiation dose [9, 10], it is important to control the dose received by nasal cavity, especially the side without tumor infiltration. However, there is no study focusing on the threshold dose to cause RICS. Therefore, we conducted this retrospective, self-controlled study to analyze association between values of dosimetric parameters and RICS onset.

## Methods

### Patient selection

A patient would be involved from clinical database of our hospital and retrospectively reviewed, if he or she had: (i) previously untreated, pathologically diagnosed NPC; (ii) complete course of radiotherapy with IMRT, from May 1st 2010 to Aug. 31st 2013; (iii) unilateral RICS diagnosed by electronic nasopharyngoscope. The cases would be excluded for: (i) prior history of any other nasal/sinusoidal disease or surgery through nasal cavity; (ii) tumor invasion directly into nasal cavities.

### Diagnostic and therapeutic procedure

Before treatment, pathologic diagnosis of each patient was achieved through nasopharyngoscope. Clinical stage was evaluated based on magnetic resonance imaging (MRI) of head and neck, computed tomography (CT) of chest and abdomen, and whole-body bone scan (or positron emission tomography). For convenience of analysis, transformation of stage into the 8th edition of the Union for International Cancer Control/American Joint Cancer Committee TNM classification were made for all the patients.

Early diseases (stage T1–2N0M0) were managed with IMRT alone. Locally advanced diseases (stage T3–4N0M0, T1-4N1-3M0) were managed with IMRT + CCT ± neoadjuvant chemotherapy (NACT). NACT before radiotherapy was repeated every 3 weeks for 2–3 cycles, with a regimen of docetaxel + cisplatin + fluorouracil or docetaxel + cisplatin. CCT of a single-agent cisplatin was repeated every week for 4–6 cycles, or every 3 weeks for 1–3 cycles. Target delineation and dose prescription of IMRT were done according to the International Commission on Radiation Units and Measurements Report 83. During radiotherapy, nasal cavities were rinsed twice daily, with sterile physiological saline.

### Follow-up

In the first 3 years after treatment, all patients were followed up every 3–6 months, with outpatient interview. At each interview, nasopharyngoscope was performed to detect recurrent primary tumor and RICS. The 22-item sinonasal outcomes test (SNOT-22) score was assessed at diagnosis of RICS. Other examinations included head-and-neck MRI, thoraco-abdominal CT and annual whole-body bone scan (or positron emission tomography). In the 4th and 5th years, follow-up was made every 6–12 months, through outpatient interview or telephone. After the 5th year, the interval of follow-up became 12 months, until death from NPC or Dec. 31st 2019, whichever came first.

### Statistical analysis

**Fig. 1:**
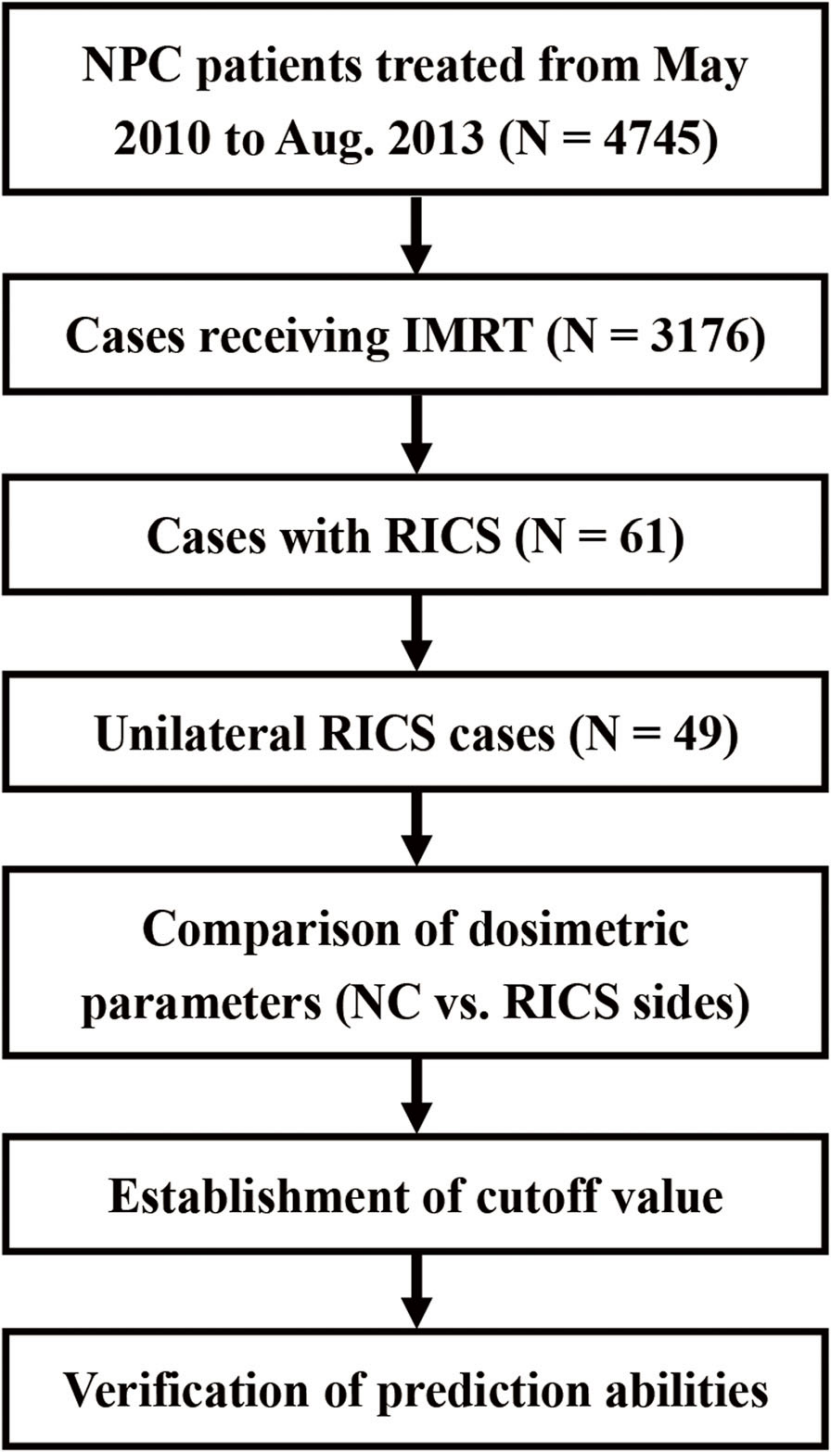
Process of this study. Abbreviations: NPC, nasopharyngeal carcinoma; IMRT, intensity-modulated radiotherapy; RICS, radiation-induced choanal stenosis; NC, normal control

Dosimetric parameters analyzed in this study included minimum point dose (Dmin), maximum point dose (Dmax), mean dose (Dmean), dose covering ≥33% volume (D33), dose covering ≥66% volume (D66), and volume receiving ≥60 Gy (V60). First, these parameters were compared between the sides of normal control (NC) and RICS, by using paired t-test. Second, receiver operating characteristic (ROC) analysis was performed in the parameters exhibiting statistical difference, with each side of nasal cavity as an individual subject, to determine their cutoff values. Third, predicting abilities of the cutoff values were checked by Chi-square test. Statistical procedure of this study referred to Fig. 1. It was completed by GraphPad Prism 6.0 (GraphPad Software, La Jolla, CA, USA) and IBM SPSS Statistics 23.0 (IBM Co., Armonk, New York, US). Difference with a two-sided *P* value of < 0.05 was regarded to be statistically significant.

## Results

### Clinical characteristics

From May 2010 to Aug. 2013, a total of 4745 NPC patients received radiotherapy in our hospital. Of those, 3176 patients were irradiated with IMRT. During the period of follow-up, 61 patients (1.9%) were found to suffer from RICS, including 49 cases with unilateral RICS. The baseline clinical profiles of the eligible patients were shown in Table 1. Continuous data is presented as median with range, and categorical data is presented as proportions (%). Among the 49 patients, there were 33 (67.3%) males and 16 (32.7%) females. The median age was 44 (range, 20–71) years old. Case numbers of T3–4 and T1–2 diseases were 40 (81.6%) and 9 (18.4%), respectively. Chemotherapy was administered in 47 (95.9%) cases. The prescribed total dose was 70.00 (66.00–74.00) Gy. RICS of right and left sides were seen in 24 (49.0%) and 25 (51.0%) cases, respectively.

**Table 1:** Baseline clinical profiles of the 49 patients with radiation-induced choanal stenosis

Characteristic	Value
Age at diagnosis (range) / years old	44 (20–71)
No. of patients by gender	
Male	33 (67.3%)
Female	16 (32.7%)
No. of patients by T stage	
T4	11 (22.4%)
T3	29 (59.2%)
T2	7 (14.3%)
T1	2 (4.1%)
No. of patients by N stage	
N3	5 (10.2%)
N2	26 (53.1%)
N1	18 (36.7%)
N0	0 (0.0%)
No. of patients by WHO pathologic subtypes	
I-II	0 (0.0%)
III	100 (100.0%)
No. of patients by prior allergic rhinitis	
Yes	3 (6.1%)
No	46 (93.9%)
No. of patients by chronic smokers	
Yes	9 (18.4%)
No	40 (81.6%)
No. of patients by treatment modes	
RT	2 (4.1%)
RT + CCT	12 (24.5%)
NACT + RT + CCT	35 (71.4%)
Prescribed total dose (range) / Gy	70.00 (66.00–74.00)
Prescribed single dose (range) / Gy	2.19 (2.06–2.34)
Actual maximum GTV dose (range) / Gy	77.22 (73.77–83.50)
Actual minimum GTV dose (range) / Gy	69.79 (65.27–72.59)
Actual mean GTV dose (range) / Gy	73.75 (65.80–78.66)
No. of patients by RICS sides	
Right	24 (49.0%)
Left	25 (51.0%)
SNOT-22 score (range)	17 (11–59)

Abbreviations: *WHO* World Health Organization, *RT* radiotherapy, *CCT* concurrent chemotherapy, *NACT* neoadjuvant chemotherapy, *GTV* gross tumor volume, *RICS* radiation-induced choanal stenosis, *SNOT-22* 22-item sinonasal outcomes test

### Follow-up outcome

**Fig. 2:**
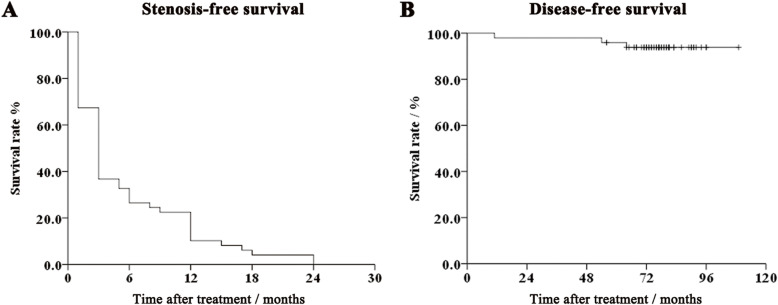
Survival curves of the 49 patients with radiation-induced choanal stenosis

The median of follow-up period was 78 (range, 56–109) months. There were 4 patients (8.2%) lost to follow-up at the 56th, 64th, 65th and 76th months, respectively. Within the 1st year after treatment, there were 44 (89.8%) RICS. No death happened in this cohort. Local recurrence and bone metastasis happened in 2 (4.1%) and 1 (2.0%) cases, respectively. Fig. 2 displayed the curves of stenosis-free and disease-free survivals, which were defined as the time period from completion of radiotherapy to onset of corresponding events.

### Dosimetric analysis

**Fig. 3:**
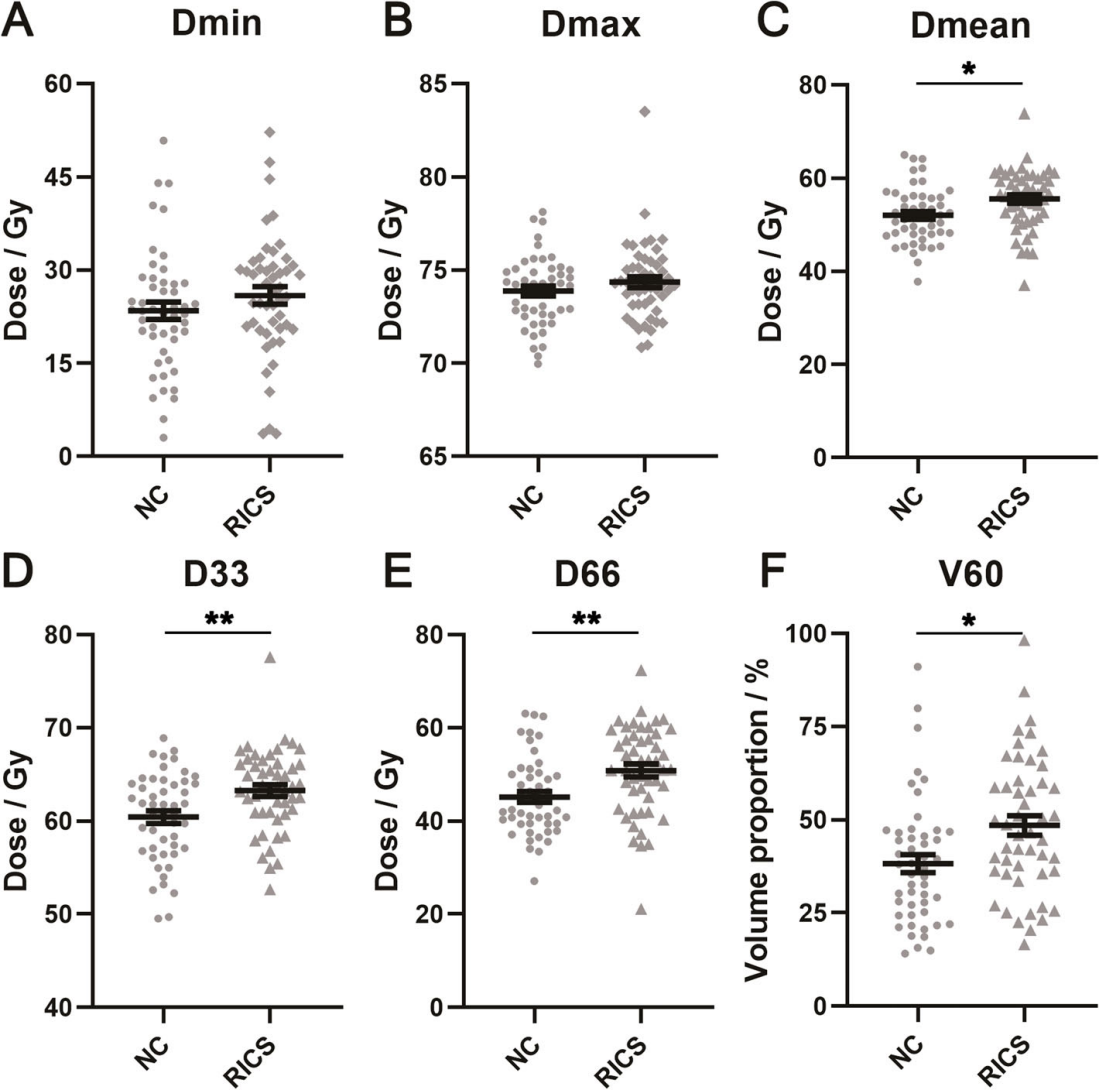
Dosimetric parameters between the nasal cavities with and without radiation-induced choanal stenosis. **a:** minimum point dose (Dmin); **b:** maximum point dose (Dmax); **c:** mean dose (Dmean); **d:** dose covering ≥33% volume (D33); **e:** dose covering ≥66% volume (D66); **f:** volume receiving ≥60 Gy (V60). Abbreviations: NC, normal control; RICS, radiation-induced choanal stenosis. * *P* < 0.05; ** *P* < 0.01

In paired t-test, RICS side of nasal cavities appeared to have higher Dmean, D33, D66 and V60, compared with NC side (Fig. 3). The means of difference (± standard error) were 3.53 ± 1.38 Gy (*P* = 0.014), 2.86 ± 0.89 Gy (*P* = 0.003), 5.65 ± 1.97 Gy (*P* = 0.006) and 10.29% ± 3.83% (*P* = 0.010). No significant difference was obtained in Dmin and Dmax.

**Fig. 4:**
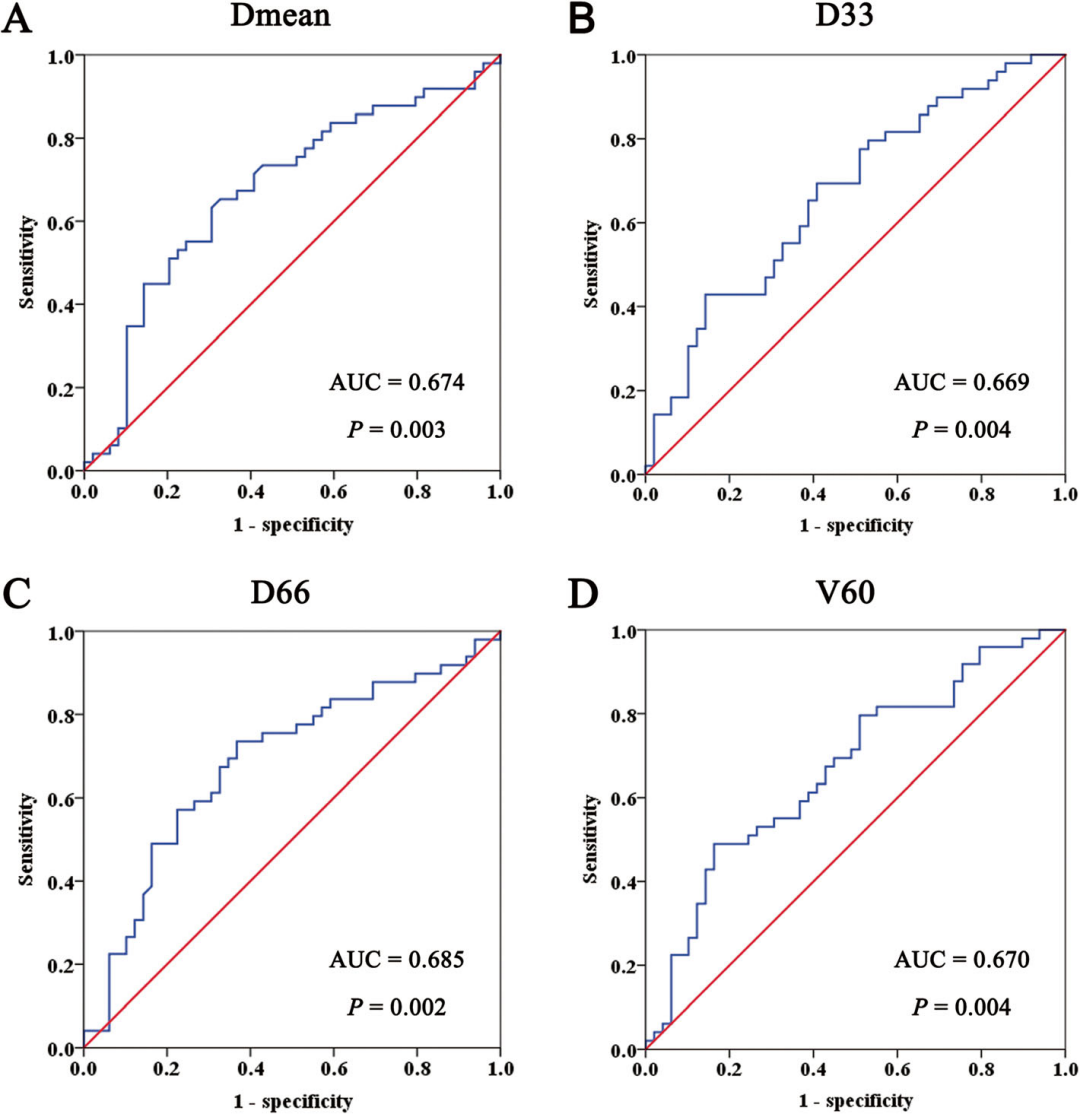
Receiver operating characteristic curves of dosimeric parameters on predicting radiation-induced choanal stenosis. **a:** mean dose (Dmean); **b:** dose covering ≥33% volume (D33); **c:** dose covering ≥66% volume (D66); **d:** volume receiving ≥60 Gy (V60). Abbreviations: AUC, area under curve

As previously described, Dmean, D33, D66 and V60 went through ROC analysis (Fig. 4). Their cutoff values for predicting RICS were 54.22 Gy, 61.96 Gy, 46.50 Gy and 48.13% (*P* values were 0.003, 0.004, 0.002 and 0.004), respectively. Then validation on predicting abilities of the cutoff values were validated (Table 2). Through Chi-square test, Dmean ≥54.22 Gy, D33 ≥ 61.96 Gy, D66 ≥ 46.50 Gy and V60 ≥ 48.13% appeared to predict RICS (*P* values were 0.001, 0.004, < 0.001 and 0.001).

**Table 2:** Prediction validation of dosimetric parameters on radiation-induced choanal stenosis

		NC	RICS	Chi-square	*P* value
Dmean / Gy	< 54.22	34 (69.4%)	18 (36.7%)	10.48	0.001 **
	≥ 54.22	15 (30.6%)	31 (63.3%)		
D33 / Gy	< 61.96	29 (59.2%)	15 (30.6%)	8.084	0.004 **
	≥ 61.96	20 (40.8%)	34 (69.4%)		
D66 / Gy	< 46.50	31 (63.3%)	13 (26.5%)	13.36	< 0.001 **
	≥ 46.50	18 (36.7%)	36 (73.5%)		
V60 / %	< 48.13	41 (83.7%)	25 (51.0%)	11.87	0.001 **
	≥ 48.13	8 (16.3%)	24 (49.0%)		

Abbreviations: *NC* normal control, *RICS* radiation-induced choanal stenosis, *Dmean* mean dose, *D33* dose covering ≥33% volume, *D66* dose covering ≥66% volume, *V60* volume receiving ≥60 Gy. ** *P* < 0.01

## Discussion

At diagnosis of RICS, the median SNOT-22 score of our cohort was 17 (range, 11–59). It is known that SNOT-22 score is the most widely accepted and best validated tool for evaluating life quality of patients with chronic rhinosinusitis. According to studies by Erskine et al. and Rudmik et al., the median SNOT-22 score in healthy population was 8 (range, 2–17). When SNOT-22 score reached 10, 30 and 50, the probability to receive endoscopic surgery increased to 37.5, 55.6 and 75.0%, respectively [11, 12]. In other words, RICS could bring seriously deteriorated life quality to NPC patients and should be avoided whenever possible. Compared with conventional 2- and 3-dimensional radiotherapy, IMRT has been proven to obviously decrease incidences of the common late toxicities in NPC patients, including xerostomia, hearing loss, trismus and temporal lobe neuropathy [13, 14]. Before this study, Shemesh et al. found 4 (4.3%) cases harboring RICS from 93 patients treated with both 2-dimensional radiotherapy and IMRT [5]. Through reviewing 3176 patients treated with IMRT uniformly, this study showed a lower incidence of RICS (1.9%). Thus, it is necessary and probable to reduce RICS through IMRT, which has a superiority in dose control.

Prior studies indicated that dosimetric parameters could predict degree of head-and-neck mucositis, which might later transformed into adhesive diseases of mucosa, such as RICS. In a study by Mazzola et al., oral mucositis of grade ≥ 2 was related to Dmean (≥ 50 Gy), Dmax (≥ 65 Gy), V45 (> 40%), V50 (> 30%) and V55 (> 20%) [9]. In a study by Musha et al., Dmax was confirmed to be associated with grade 2–3 oral mucositis. Moreover, the threshold value was discovered to vary for different sites. For palate and tongue, the figures were 43.0 and 54.3 Gy, respectively [10]. In literrature, there was merely one study directly reporting the correlation between radiation dose and nasal mucositis. Riva et al. discovered that elevated Dmean and D2 were associated with a higher incidence of nasal mucositis [15]. But the severity was not discussed in that study. And the sample size of this study was quite small.

This study made the first approach to how radiation dose affected RICS. Here we revealed that nasal cavity with RICS underwent greater radiation dose than that without RICS. Higher dosimetric parameters were observed in the RICS side, including Dmean (55.54 ± 0.93 vs. 52.01 ± 0.86 Gy, *P* = 0.014), D33 (63.29 ± 0.63 vs. 60.43 ± 0.68 Gy, *P* = 0.003), D66 (50.85 ± 1.37 vs. 45.20 ± 1.19 Gy, *P* = 0.006) and V60 (48.50 ± 2.60% vs. 38.21 ± 2.41%, *P* = 0.010). Unexpectedly, Dmax of the RICS side was similar to that of the NC side (74.35 ± 0.30 vs. 73.88 ± 0.27, *P* = 0.273). Next, we further established cutoff value of these parameters for predicting RICS. Patients with Dmean ≥54.22 Gy, D33 ≥ 61.96 Gy, D66 ≥ 46.50 Gy and V60 ≥ 48.13% emerged to be at a higher risk to develop RICS. Among these parameters, D66 had the best sensitivity (73.5%) and negative predictive value (70.5%). V60 had the best specificity (83.7%) and positive predictive value (75.0%). This study provided a reference for dose control of nasal cavity without invasion or approximation of tumor. Its results were based on a cohort with a relatively large sample size. And self-controlled design averted biases brought by imbalance of baseline characteristics. These were the strengths of our study.

It was noteworthy that in this study, 89.8% of RICS happened within the 1st year after radiotherapy. Similarly, the 4 cases presented by Shemesh et al. had RICS in 2–12 months [5]. And in the 6 cases presented by Ku et al., the mean time to RICS was 10.5 months [8]. So it could be inferred that this period was critical for controlling nasal mucositis and RICS formation. Up to now, saline rinsing was the only effective method supported by strong evidences to manage chronic nasal mucositis [16]. On basis of our results, nasal saline rinsing was suggested to be carried out during radiotherapy and until at least 1 year after. Recently, Canakci et al. found in rat models that local application of *Nigella sativa* oil could relieve radiation-induced erosion of nasal mucosa [17]. Duan also found in pig models that mesenchymal stem cells from human umbilical cord could alleviate mucosal edema caused by radiotherapy and improve mucus clearance [18]. These methods might become treatment choices of nasal mucositis in future. In addition, smoking and poor nutritional condition was demonstrated to increase serious severe oral mucositis [19, 20]. Quitting smoking and nutritional support might be helpful for prevention of RICS.

Indeed, this study had 3 main disadvantages, due to rarity of RICS. First, it was a retrospective study. Second, it was an observational study without active intervention in radiation dose. Third, it had no verification in an independent cohort. Hence, the results of this study needed to be popularized with caution and externally validated by prospective randomized controlled trials.

## Conclusion

Dmean, D33, D66 and V60 of nasal cavity could act as predictors of RICS. For ameliorating life quality of NPC patients, the values of these dosimetric parameters should be controlled whenever possible.

## Data Availability

The data that support the findings of this study are available from the corresponding author upon reasonable request.

## Abbreviations

NPC: Nasopharyngeal carcinoma
IMRT: Intensity-modulated radiotherapy
CCT: Concurrent chemotherapy
RICS: Radiation-induced choanal stenosis
MRI: Magnetic resonance imaging
CT: Computed tomography
NACT: Neoadjuvant chemotherapy
SNOT-22: 22-item sinonasal outcomes test
Dmin: Minimum point dose
Dmax: Maximum point dose
Dmean: Mean dose
D33: Dose covering ≥33% volume
D66: Dose covering ≥66% volume
V60: Volume receiving ≥60 Gy
NC: Normal control
ROC: Receiver operating characteristic
